# Photoacoustic and absorption spectroscopy imaging analysis of human blood

**DOI:** 10.1371/journal.pone.0289704

**Published:** 2023-08-04

**Authors:** Wei-Yun Tsai, Stephan Breimann, Tsu-Wang Shen, Dmitrij Frishman

**Affiliations:** 1 Department of Bioinformatics, Wissenschaftszentrum Weihenstephan, Technische Universität München, Freising, Germany; 2 Department of Automatic Control Engineering, Feng Chia University, Taichung, Taiwan; 3 Master’s Program Biomedical Informatics and Biomedical Engineering, Feng Chia University, Taichung, Taiwan; Universita degli Studi di Milano-Bicocca, ITALY

## Abstract

Photoacoustic and absorption spectroscopy imaging are safe and non-invasive molecular quantification techniques, which do not utilize ionizing radiation and allow for repeated probing of samples without them being contaminated or damaged. Here we assessed the potential of these techniques for measuring biochemical parameters. We investigated the statistical association between 31 time and frequency domain features derived from photoacoustic and absorption spectroscopy signals and 19 biochemical blood parameters. We found that photoacoustic and absorption spectroscopy imaging features are significantly correlated with 14 and 17 individual biochemical parameters, respectively. Moreover, some of the biochemical blood parameters can be accurately predicted based on photoacoustic and absorption spectroscopy imaging features by polynomial regression. In particular, the levels of uric acid and albumin can be accurately explained by a combination of photoacoustic and absorption spectroscopy imaging features (adjusted R-squared > 0.75), while creatinine levels can be accurately explained by the features of the photoacoustic system (adjusted R-squared > 0.80). We identified a number of imaging features that inform on the biochemical blood parameters and can be potentially useful in clinical diagnosis. We also demonstrated that linear and non-linear combinations of photoacoustic and absorption spectroscopy imaging features can accurately predict some of the biochemical blood parameters. These results demonstrate that photoacoustic and absorption spectroscopy imaging systems show promise for future applications in clinical practice.

## Introduction

Photoacoustic and absorption spectroscopy imaging are emerging non-invasive imaging modalities with a broad spectrum of potential biomedical applications. In photoacoustic imaging, a tissue sample is irradiated with a laser pulse. Depending on the optical absorption properties of the chromophore, the absorbed photon energy is converted to heat and generates an acoustic wave, which is detected by an ultrasonic transducer [1]. In absorption spectroscopy imaging, biomolecules absorb light pulses emitted by light-emitting diodes (LED). The scattered light is detected by a balanced detector and the signal is captured and processed by a digital oscilloscope [2].

Both photoacoustic and absorption spectroscopy imaging are easy-to-use, low-cost, non-ionizing and non-radioactive methods capable of detecting absorption spectroscopy contrast in a variety of biological tissues [3, 4]. Here, we apply photoacoustic/ absorption spectroscopy imaging systems to detect biochemical parameters from blood samples in vitro. Blood reflects the status of internal organs and tissues throughout the body and is therefore important in detecting, monitoring, and treating many diseases. Photoacoustic signals have been applied in many blood-related studies [5], such as the observation of size and shape of red blood cells [6–9], measurement of blood glucose concentration [10, 11], early malaria diagnosis [12, 13], diagnosis of bacteria in the blood [14], detection of circulating tumor cells [15, 16], investigation of hemoglobin oxygen and carboxyhemoglobin saturation [17, 18], bilirubin monitoring [19], and pharmaceuticals monitoring [20–22]. In particular, light scattering by blood cells can be effectively used to assess the general state of health and catch the early signs of multiple diseases [23]. Changes of glucose concentration can be used to diagnose diabetes [24], urea, creatinine, eGFR, and uric acid levels are related to renal function [25], total cholesterol, triglycerides, as well as high-density and low-density lipoproteins have relevance to cardiovascular disease [26, 27]. Alanine aminotransferase and albumin are indicators of liver and bile duct function [28], while Na, K, and Cl levels are sensitive to hypertension [29, 30]. Those compounds may provide different spectrums of ultrasound echoes in photoacoustic measurements.

In this study, we analyzed photoacoustic and absorption spectroscopy imaging signals with both the time domain and frequency domain. The time domain graph displays a signal over time, whereas the frequency domain graph displays how much signal is in each specific frequency band over a range of frequencies. The time domain graph displays the changes of the signal over time, whereas the frequency domain graph reflects the distribution of the signal over frequency bands. The time domain graph can thus be directly used to observe the features of the waveform without transformation, while the frequency domain graph needs to be transformed. The frequency domain analysis reveals some additional features, such as PASA slope, midband fit, and intercept. Many research groups attempted to quantify the histologic information by specific features of photoacoustic and absorption spectroscopy imaging signals, both in the time domain (amplitude [10], rising and descending trend [31], area [10]) and in the frequency domain (slope, Midband fit, intercept [3], full width at half maximum (FWHM) [32], prominence, and area [10]). However, a comprehensive feature analysis of photoacoustic and absorption spectroscopy imaging signals applied to biochemical parameters has been lacking so far. In the present study, we investigated the connection between 31 imaging features from the time and frequency domains and a broad spectrum of biochemical parameters of human blood. We have also constructed polynomial regression models to predict biochemical parameters based on imaging signal features.

## Materials and methods

### Blood samples

Sample collection and analysis followed the protocol approved by the Tzu Chi university. This study was performed according to the regulation of the Institution Review Board of the Tzu Chi Hospital (Taiwan), ID number IRB105-137-B. All participants received a written informed consent containing a detailed explanation of the human experiments and signed the informed consent before the experiments. All experiments were performed in accordance with relevant guidelines and regulations. Photoacoustic imaging, absorption spectroscopy imaging, and biochemical measurements were performed at the Tzu Chi Hospital on blood drawn from 14 individuals (6 male and 8 female) aged 30–71. There were 12 individuals for which both photoacoustic and absorption spectroscopy imaging measurements were performed, 1 individual with only a photoacoustic measurement and 1 with only an absorption spectroscopy imaging measurement.

### Biochemical blood parameters

For each patient, the total of 19 biochemical parameters were measured: ALT (alanine aminotransferase), TP (total protein), ALB.BCG (albumin), GLO (globulin), BUN (blood urea nitrogen), UA (uric acid), CRE (creatinine), TCH (total cholesterol), TG (triglyceride), GLU.AC (glucose), eGFR (estimated glomerular filtration rate), Na, K, Cl, Ca, Fe, TIBC (total iron binding capacity), HDL.C (high-density lipoprotein), LDL.C (low-density lipoprotein).

### Photoacoustic imaging by laser pulses

**Fig 1:**
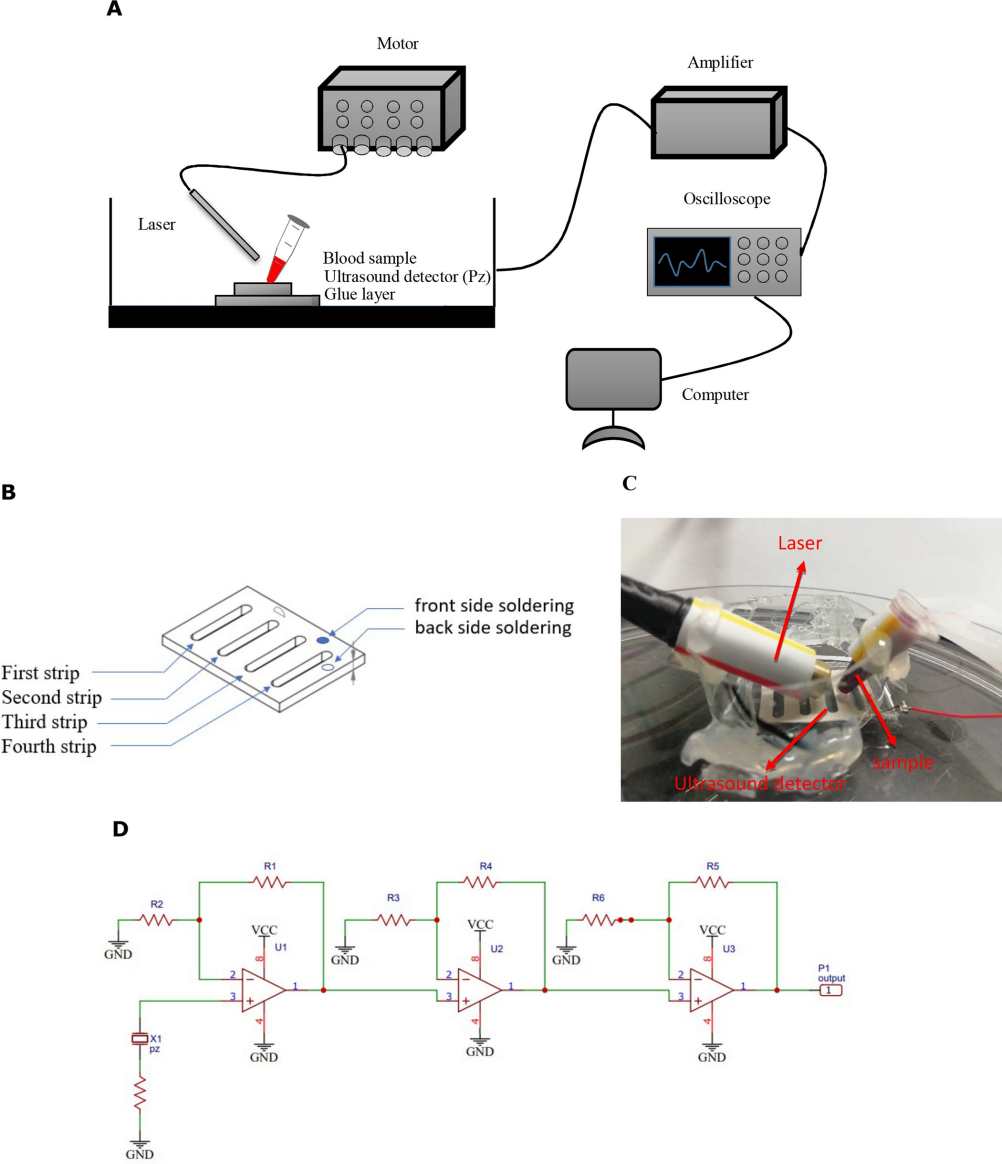
Schematic diagram of the photoacoustic imaging system and the placement of class IIIB laser, ultrasound detector and blood sample. (A) The blood sample is irradiated with a laser pulse and the absorbed photon energy is converted to heat and generates an acoustic wave, which is detected by an ultrasonic detector. The signal is amplified by an amplifier and recorded on a digital oscilloscope. Finally, the computer is used to process the features and extract the features from the signal. (B) The structure of the piezoelectric ceramic piece. (C) The placement of the blood sample, the class IIIB laser and the ultrasonic detector. (D) The amplifier circuit diagram. (This figure has been updated).

The experimental setup for photoacoustic imaging consists of the following parts (Fig 1):

1. Class IIIB laser (LECC, Taiwan) with the wavelength of 658 nm, which is within the traditional optical window used for biological tissues [33, 34], output power of 80 mW, pulse width of 0.5 ms, energy of 32.5 mJ, sampling duration of 0.002 ms, and pulse repetition rate of 1k Hz. The total time of the laser activation is 0.5 ms.
2. The power supply (MOTECH LPS-305) as a tool to control the laser.
3. An ultrasound detector (piezoelectric sensor) for detecting laser-induced photoacoustic signals and converting them into electrical signals. We used the ultrasound transducer (Unictron Technologies Crops, Taiwan) with the resonant frequency 3.20MHz ± 3‰, resonant impedance less than 0.65 Ω, and electromechanical coupling coefficient larger than 49%. The piezoelectric ceramic piece has a length of 21.9 mm and a width of 15.9 mm with four strip holes of 12 mm in length and 2 mm in width, as shown in Fig 1B.
4. A low noise amplifier (LNA), which can amplify very low-power signals without significantly degrading their signal-to-noise ratio, was connected to the ultrasound transducer. The gain of the LNA is 271 times and the input referred noise of the operational amplifier (Texas instruments Incorporated OPA847, USA) is 1 nV/√Hz.
5. A digital oscilloscope (TDS 2024C, Tektronix) for recording PA signals from blood samples with the resampling rate of 500000.
6. A computer for extracting and processing features from the averaged PA signal.
7. Plastic containers (Sorenson BioScience Inc., 0.2 mL PCR tubes with attached caps, Salt Lake City, UT, USA) for holding samples on an ultrasonic detector, sealed with glue in order to minimize instability from external factors. The laser was focused on the tip position of the plastic containers but not on the ultrasound detector to avoid affecting the photoacoustic signal.

The voltage of the photoacoustic signals generated by laser pulses was in the approximate range between -1 V and 0.5 V.

The beginning voltage of the signals was negative. In order to make measurements more easily comparable between samples we shifted the photoacoustic signal values such that they start from 0 and lie in the approximate range between 0–1.5 a.u.

### Absorption spectroscopy imaging by light emitting diodes

**Fig 2:**
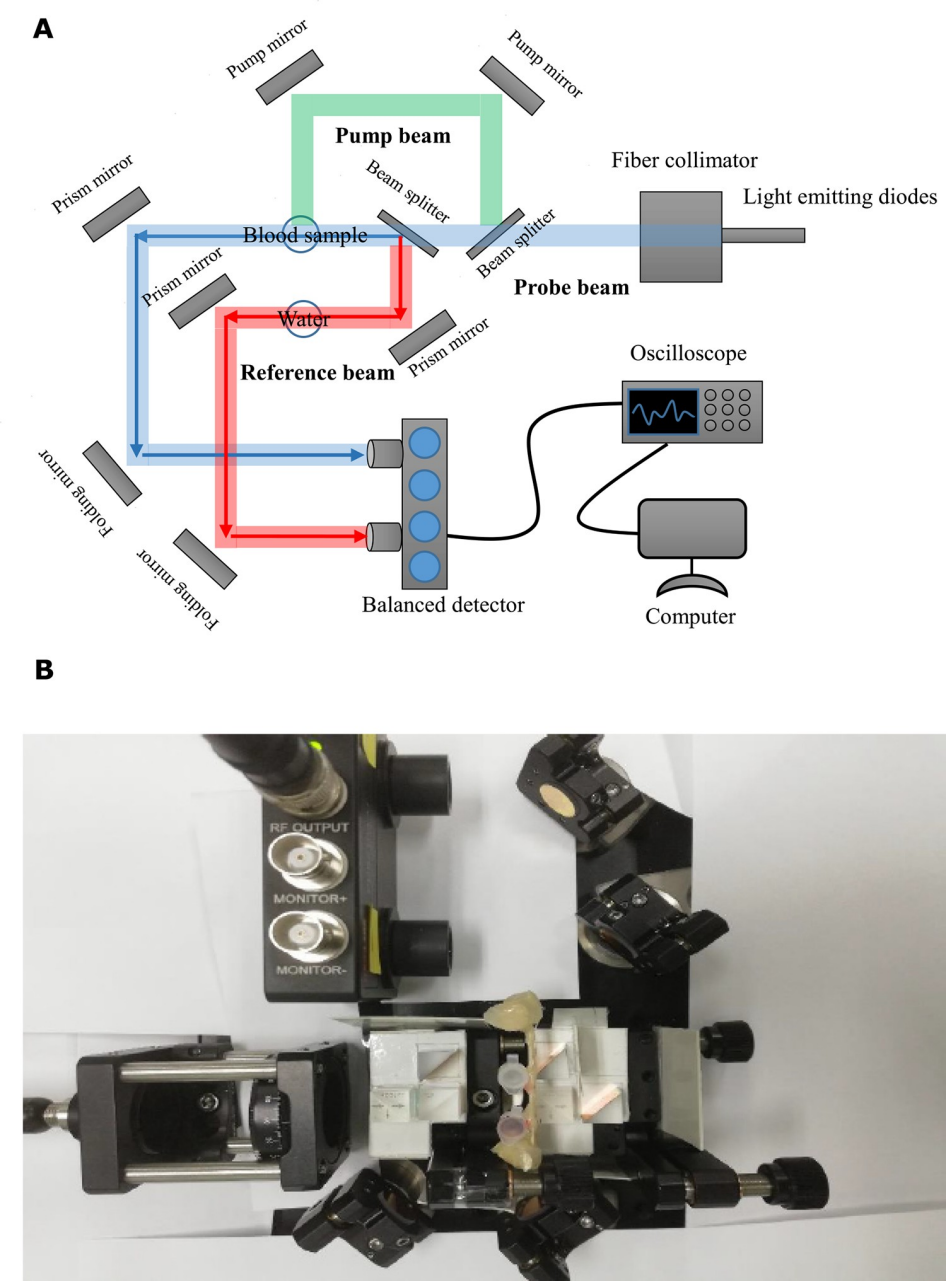
Schematic diagram of the absorption spectroscopy imaging system with light emitting diodes, the path of the light from the light emitting diode, and the experimental setup for the absorption spectroscopy imaging system. (A) The blood sample absorbs light pulses emitted by light–emitting diodes (LED). The scattering of light is detected by a balanced detector. Finally, the signals are captured and processed by digital oscilloscope and the computer is used to process the features and extract the features from the signals. (B) The placement of the components in the optical imaging system. (This figure has been updated).

The experimental setup for absorption spectroscopy imaging consists of the following parts (Fig 2):

1. Light emitting diodes (Kolin, Taiwan) generating 0.45 ms pulse width at 650±10nm, coupled into the setup using an optical fiber.
2. The Thorlabs SKSAS (Saturated Absorption Spectroscopy Systems) kit, which includes a fiber collimator, prisms, mirrors, and a balanced detector. A fiber collimator (F220FC-780, Thorlabs) collimates the light, which is then passed through a half wave plate and a polarizing beam splitter cube in order to split off power for the “pump” beam. The remaining light is split into two parallel beams, the “probe” beam, and the “reference” beam, which pass through the blood sample and water, respectively. The experiment must ensure that the path lengths (blue and red arrows) of these two beams are the same. The two photodiodes of the balanced detector (PDB210A) with a power supply (LDS1212, Thorlabs) of 115 mA detect the probe and reference beams.
3. A digital oscilloscope (TDS 2024C, Tektronix) attached to the radio frequency OUTPUT connector of the balanced detector to record signals from samples.
4. A computer for processing and extracting features from the averaged PA signal.
5. Plastic containers (Sorenson BioScience Inc., 0.2 mL PCR Tubes with Attached Caps, Salt Lake City, UT, USA) for holding samples and water. The light with a beam diameter 1.5 mm was focused on the tip position of the plastic containers.

The voltage of the absorption spectroscopy signals generated by light pulses was approximately in the range between -100 V to 20 V. The beginning voltage of the signals was negative. In order to make measurements more easily comparable between samples we shifted the range of absorption spectroscopy signal values to start from 0. Therefore, the intensity of the absorption spectroscopy signals generated by light pulses was approximately in the range between 0 a.u. to 120 a.u.

### Photoacoustic and absorption spectroscopy signal features

**Fig 3:**
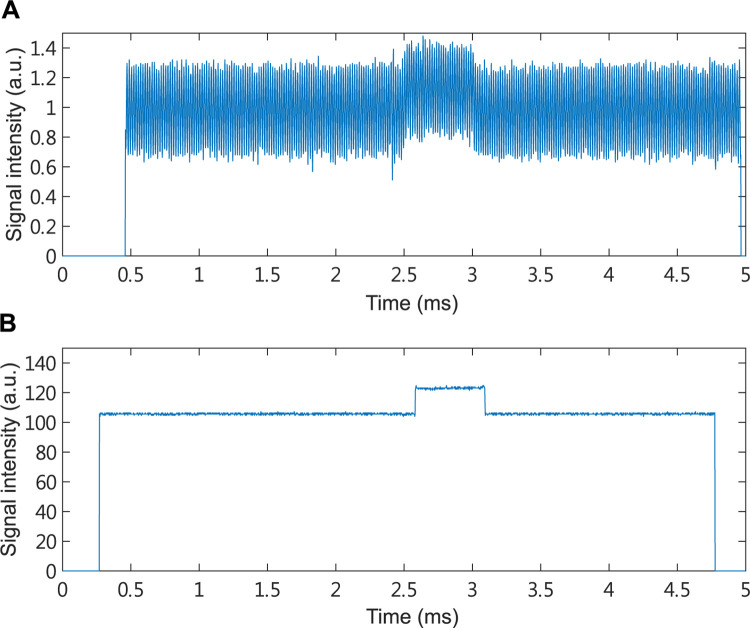
Example of signals from photoacoustic (A) and absorption spectroscopy (B) experiments. In all experiments 2500 samples of the signal were collected over 5 milliseconds with a sampling interval of 0.002 milliseconds.

**Fig 4:**
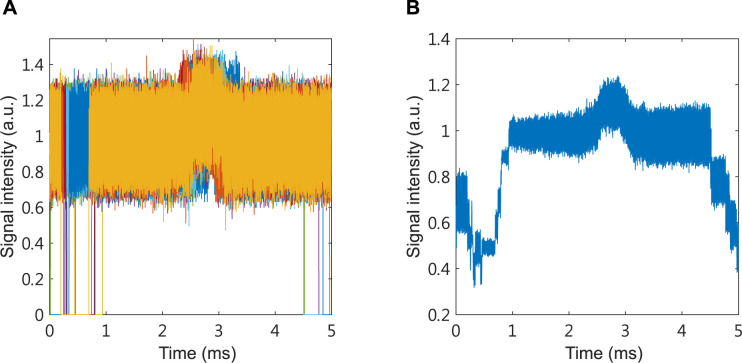
Pre–and post–averaging of PA signal traces from laser samples. (A) Each experiment includes 2500 signal samples in 5 milliseconds with a sampling interval of 0.002 milliseconds. The photoacoustic experiment was repeated 10 times for each individual. Different colors represent 10 repetitions of photoacoustic experiments. (B) To reduce the noise, the signal intensity at each time point was averaged.

**Fig 5:**
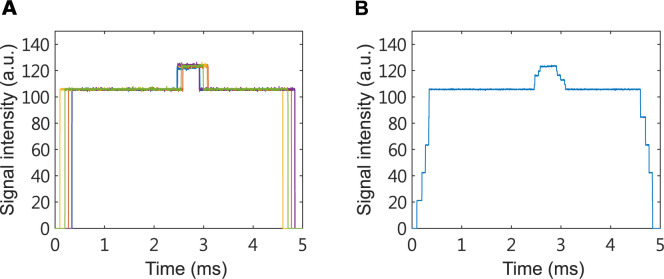
Pre–and post–averaging absorption spectroscopy signal traces from light samples. (A) Each experiments includes 2500 signal samples in 5 milliseconds with a sampling interval of 0.002 milliseconds. The absorption spectroscopy experiment was repeated 5 times for each individual. Different colors represent 5 repetitions of absorption spectroscopy experiments. (B) To reduce the noise, we averaged the signal intensity at each time point.

In order to make measurements more easily comparable between samples we shifted the range of both photoacoustic (Fig 3A) and absorption spectroscopy (Fig 3B) signal values to start from 0 a.u. In all experiments 2500 samples of the signal were collected over 5 milliseconds with a sampling interval of 0.002 milliseconds. For each individual, photoacoustic and absorption spectroscopy experiments were repeated 10 and 5 times, respectively (Figs 4A and 5A). At each time point the signal intensity was averaged over the number of experiments to reduce the noise (Figs 4B and 5B). The trigger has an error in the switching time of each experiment, and the error time is about 0.5 ms. To reduce analysis error, we only use signals in the steady state for further analysis. Subsequently, the total of 31 time and frequency domain features were extracted from the signal, summarized in Table 1.

**Table 1:** The list of imaging features.

Feature type	Feature category	Features in each category
**Time domain**	Amplitude	Peak-to-Peak Amplitude, Amplitude of the positive peak, Amplitude of the negative peak
	Rising trend	Positive slope
	Descending trend	Negative slope
	Area	Time domain area
**Frequency domain**	PASA slope	[0–1 MHz], [1–2 MHz], [2–3 MHz], [0–3 MHz]
	Midband fit	[0–1 MHz], [1–2 MHz], [2–3 MHz], [0–3 MHz]
	Intercept	[0–1 MHz], [1–2 MHz], [2–3 MHz], [0–3 MHz]
	FWHM	[0–0.5 MHz], [0.5–1 MHz], [1–1.5 MHz], [1.5–2 MHz], [2–2.5 MHz], [2.5–3 MHz]
	Prominence	[0–0.5 MHz], [0.5–1 MHz], [1–1.5 MHz], [1.5–2 MHz], [2–2.5 MHz], [2.5–3 MHz]
	Area	Frequency-domain area

### Time domain features

**Fig 6:**
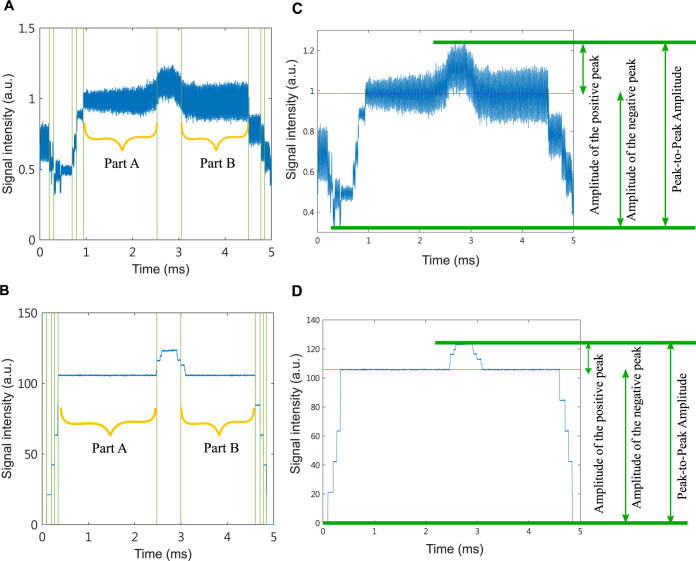
Definition of two windows and the amplitude–related features in the photoacoustic and absorption spectroscopy signal. (A) and (B) are signals generated by photoacoustic and absorption spectroscopy systems, respectively. The green lines indicate the top 10 abrupt changes determined by the *findchangepts* function in MATLAB. Part A and part B are located on the left and the right sides of the window containing the largest signal intensity. (C) The definition of peak–to–peak amplitude and amplitude of the positive and negative peak were according to the average intensity (red line). (D) The definition of peak–to–peak amplitude and amplitude of the positive and negative peak were according to the average intensity (red line).

Following previous research [4, 23], we calculated 3 types of amplitude features: peak-to-peak amplitude and amplitude of the positive and negative peak. The first step in calculating the average amplitude was to find the top 10 abrupt changes in the signal using the *findchangepts* [35] function in MATLAB (Fig 6A and 6B). Subsequently we defined the windows on the left (part A) and right (part B) sides of the window containing the largest signal intensity and averaged the intensity over these two windows. Peak-to-peak amplitude was calculated as the range between the maximum and minimum intensity, while the amplitude of the positive and negative peaks is the difference between the maximum and the average intensity as well as between the average and minimum intensity, respectively (Fig 6C and 6D).

### Positive and negative slopes

**Fig 7:**
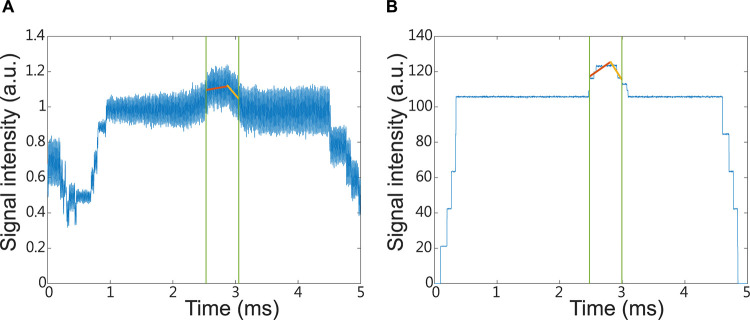
Illustration of the positive and negative slopes. (A) and (B) are signals generated by photoacoustic and absorption spectroscopy systems, respectively. The green lines indicate the end of part A and the beginning of part B, respectively. The red and yellow lines indicate the positive and negative slopes, respectively.

These measures have been previously applied for photoacoustic detection of protein coagulation [31] and for analyzing the hemodynamic response by functional near-infrared spectroscopy [36]. We conducted a search for slopes in the region bounded by the end of part A and the beginning of part B (see above and Fig 7). The region to calculate the positive slope is from the end of part A to the maximum of the signal intensity. The region to calculate the negative slope is from the maximum of the signal intensity to the beginning of part B. The relationship between the intensity of the optical absorption spectra *y* and the time *x* was modeled in form of a linear regression y = bx+a, where n is the signal sample size and the intercept a and the slope b were calculated as

$$a=\frac{(\sum y)(\sum x^{2})-(\sum x)(\sum xy)}{n(\sum x^{2})-{\left(\sum x\right)}^{2}}$$ (1)

$$b=\frac{n(\sum xy)-(\sum x)(\sum y)}{n(\sum x^{2})-{(\sum x)}^{2}}$$ (2)

### Frequency domain features

Frequency domain features are suitable for evaluating the morphology of optically absorbing objects [37] and investigating the microscopic architecture of biological tissues [38]. The time to frequency domain conversion of the signal was affected by the Welch’s method (as implemented by the *pwelch* function in MATLAB) [39, 40]. Power spectra were estimated by dividing the time signal into successive Hamming windows of 1/8 of the total signal length overlapping by 50%, forming the periodogram for each block, and then averaging the periodograms over time.

### PASA slope, midband fit and intercept

**Fig 8:**
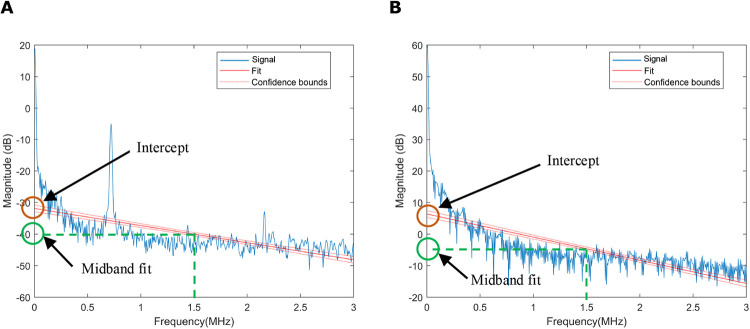
Frequency domain analysis. The PASA slope (red lines), the values of intercept (orange circles) and midband fit (green circles) were showed in photoacoustic (A) and absorption spectroscopy (B) signals, respectively.

**Fig 9:**
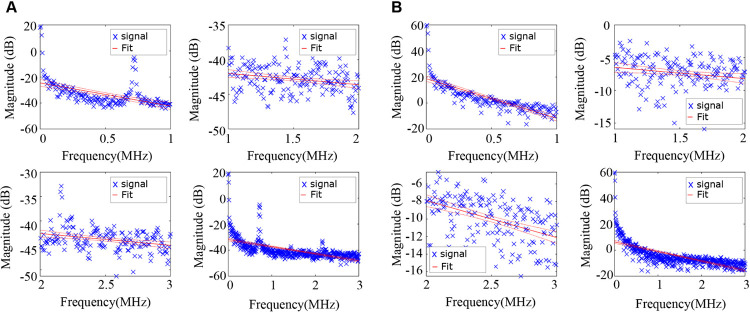
Different frequency bands used for frequency domain analysis. (A) and (B) are signals generated by photoacoustic and absorption spectroscopy systems, respectively. In order to differentiate between tissues more precisely, we divided the signal into 4 frequency bands (0–1 MHz,1–2 MHz, 2–3 MHz, and 0–3 MHz). The red lines indicate the PASA slopes.

Photoacoustic spectrum analysis (PASA) is a non-invasive technique to investigate the microstructures in biological tissues [41]. It has been applied to assessing the Gleason scores of prostate cancers [38], quantifying the lipid content of liver [42], and differentiating between tumor types [43]. Power spectra were fit by a linear regression model using the *fitlm* function in MATLAB and then the slope, intercept and midband fit values were determined (Figs 8 and 9), which reflect the magnitude of high vs low frequency components, the zero frequency, and the average magnitude of the power spectrum, respectively [41]. These three parameters have been shown to correlate with the microstructural properties of tissues [3, 44]. In order to differentiate between tissues more precisely, we divided the signal into 4 frequency bands [45] (0–1 MHz,1–2 MHz, 2–3 MHz, and 0–3 MHz) (Fig 9).

### Prominence and FWHM

**Fig 10:**
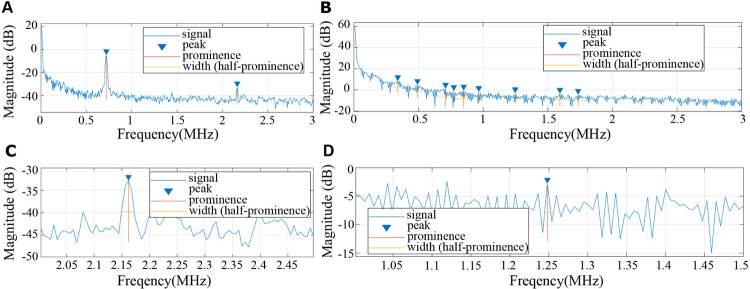
The definition of Prominence and FWHM in frequency domain analysis from photoacoustic signals and absorption spectroscopy signals. (A) Peaks of the photoacoustic signals with a prominence larger than 10 are identified. (B) A prominent peak of the photoacoustic signals in the 2–2.5 MHz band. (C) Peaks from absorption spectroscopy signals with a prominence larger than 10 are identified. (D) A prominent peak of the absorption spectroscopy signals in the 1–1.5 MHz band.

Prominence is the height of a peak relative to the lowest contour line around that peak. For peaks with significant prominence, we also computed full width at half maximum (FWHM), the distance between the points where the magnitude is half of the maximal value. FWHM is a parameter describing pulse waveforms which was previously used to characterize blood viscosity in frequency-resolved photoacoustic measurements [32]. FWHM and prominence were calculated using the *findpeaks* function in MATLAB. We utilized prominence = 10 as a threshold, then only selected peaks with the maximum prominence in each frequency bands (0–0.5 MHz, 0.5–1 MHz, 1–1.5 MHz, 1.5–2 MHz, 2–2.5 MHz, and 2.5–3 MHz) (Fig 10).

#### Area under the photoacoustic signal waveform and frequency spectrum

Area-based features have been shown to correlated with glucose concentration in whole blood samples [10]. We calculated the area under the signals in the time and frequency domain by the *trapz* function in MATLAB.

### Correlation analysis

We computed Spearman’s correlations between biochemical and imaging features and assessed their significance by the R function *rcorr* [46]. Pairs of features with the absolute correlation coefficient > 0.5 and p-values ≤ 0.05 were considered to be significantly associated.

### Model building

We investigated the performance of photoacoustic/absorption spectroscopy imaging features in predicting biochemical blood parameters based on polynomial regression, as implemented in the *lm* function of the R project. Only significantly associated features were considered, based on Spearman’s correlation. To test for multicollinearity between explanatory variables [47, 48] we entered biomedical parameters and imaging features into a polynomial regression model with degree 1. Multicollinearity was diagnosed based on the variance inflation factor (VIF) calculated using the *vif* function from the “car” R package [49]. Following the approach described in [50], the feature with the maximal VIF value (VIF>10) was removed and the diagnostic process was continued until only features with VIF ≤ 10 remained.

Next, we determined whether imaging features had a linear or a non-linear relationship with the blood parameters. Validation of the linear model assumptions [51] was performed using the *gvlma* function from the “gvlma” R package. This function performs a single global test to assess the linear model assumption as well as four specific directional tests designed to detect skewness, kurtosis, a nonlinear link function, and heteroscedasticity. For the global test the null hypothesis is that the relationship between two features is linear. The null hypothesis for the skewness and kurtosis tests is that the residuals are normally distributed. The null hypothesis of the link function is that the variable is continuous. Finally, for heteroscedasticity the null hypothesis is that the variance of the model residuals remains constant. We also utilized the Durbin-Watson test, as implemented in the “car” R package [49], to determine the independence of error terms, the null hypothesis being that the residuals are independent. If *all* the above null hypotheses were not rejected, an imaging feature was defined as linearly related with a biochemical parameter, otherwise a non-linear relationship was assumed.

Subsequently, linear relationships were fitted using polynomial regression models with a degree 1, while for non-linear relationships degrees 2 or 3 were chosen based on the Bayesian information criterion (BIC) [52], calculated as

$$BIC=\ln\left(n\right)\;k-2ln\;(\hat{L})$$ (3)

where n is the sample size, k is the number of parameters to be estimated, and $\hat{L}$ is the maximize value of the likelihood function of the model. The model with the lowest BIC value was selected.

The quality of the fit was assessed based on the adjusted R-squared and the p-value of the F-statistic. Adjusted R-squared values were computed using the following equation:

$$Adjusted\;R^{2}=1-\frac{\left(1-R^{2})(n-1\right)}{n-p-1}$$ (4)

with the sample R-squared given by:

$$R^{2}=1-\frac{\sum_{i=1}^{n}{\left(y_{i}-y_{i}^{⋀}\right)}^{2}}{\sum_{i=1}^{n}{\left(y_{i}-y_{mean}\right)}^{2}}$$ (5)

where n is the number of data points, y is the actual value, *y*^ is the predicted value, *y*_*mean*_ is the mean of y and p is the total number of explanatory variables in the model.

## Results

### Overview of the study

**Fig 11:**
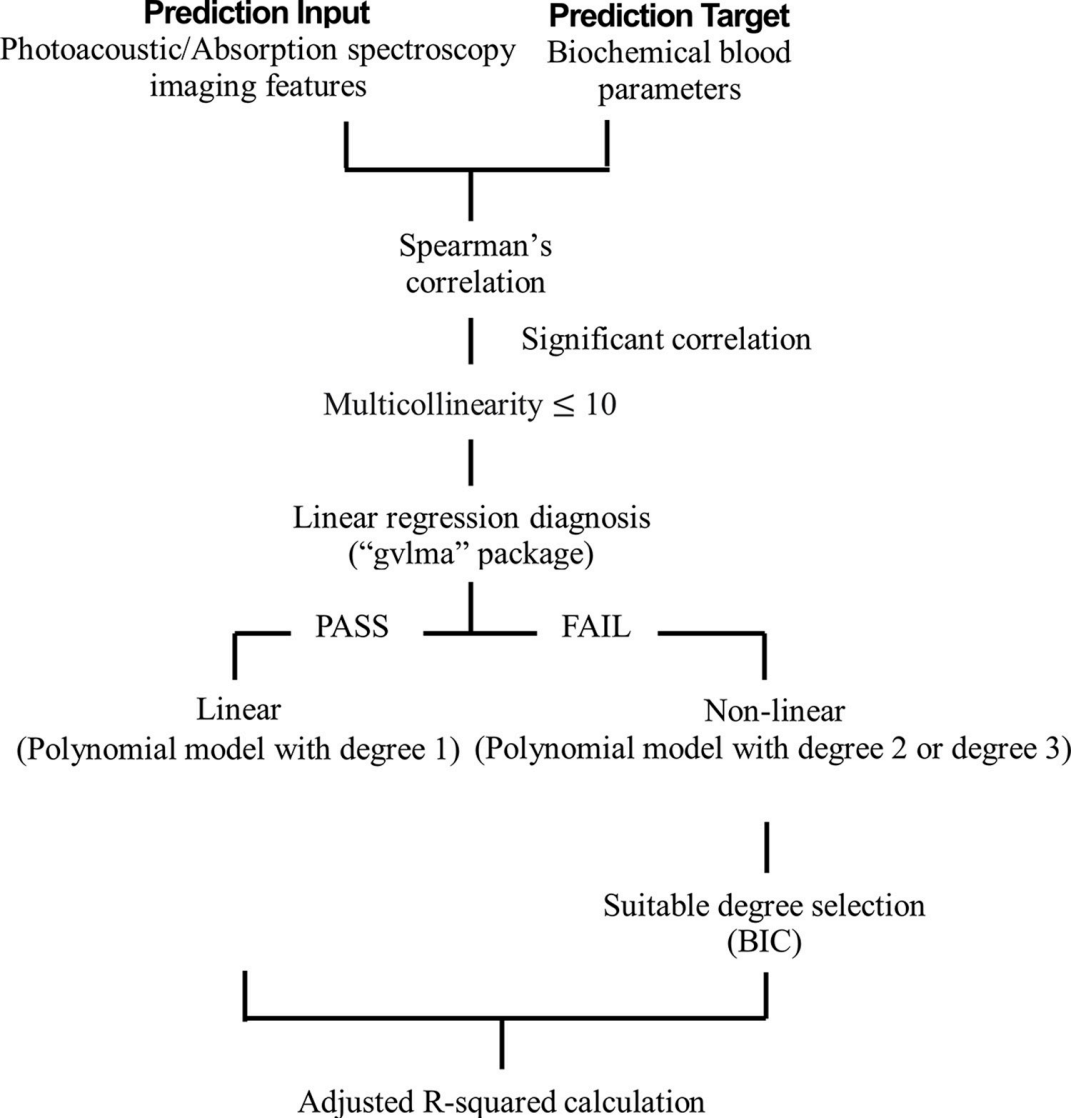
Workflow of the study. The study was based on different conditions to construct the polynomial models, which include Spearman’s correlation analysis, multicollinearity, linear regression diagnosis, determination of linearly or non–linearly, degree selection. Polynomial models were constructed with these conditions and the adjusted R–square of the models was calculated.

The goal of the study was to investigate to which extent photoacoustic and absorption spectroscopy imaging features are correlated with and predictive of biochemical parameters (Fig 11). Significantly correlated features were tested for multicollinearity. Subsequently it was determined whether imaging features were related to the biochemical parameters linearly or non-linearly. In the former case, polynomial models with degree 1 were used, while in the latter case the choice between polynomial models with degree 2 or 3 was guided by the Bayesian information criterion. The quality of the fit was assessed based on adjusted R^2^. See Materials and Methods for a detailed explanation of experimental procedures, imaging and biochemical parameters, and statistical models.

### Feature selection and correlation analysis

**Fig 12:**
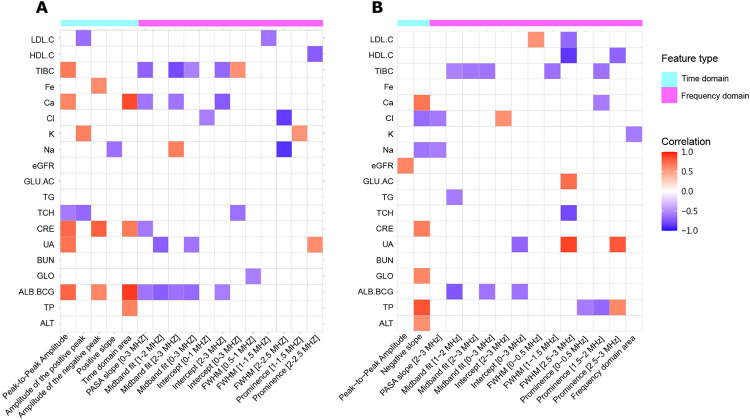
Heatmap representation of statistically significant (p–value≤0.05, r_s_ > 0.5) associations between imaging features (columns) and biochemical blood parameters (rows). (A) Photoacoustic signals, (B) Absorption spectroscopy signals.

We found that 17 out of 31 photoacoustic imaging features were significantly correlated with at least one biochemical blood parameter (Fig 12A). Time domain features (peak-to-peak amplitude, amplitude of the positive peak, and amplitude of the negative peak, positive slope and the area under the signal waveform of time domain) tend to exhibit positive correlation while frequency domain features (PASA slope, Midband fit, Intercept, FWHM, and Prominence) are more frequently negatively correlated. The results for the absorption spectroscopy imaging features (Fig 12B) were qualitatively similar, with two time domain features (Peak-to-Peak Amplitude and Negative slope) and thirteen frequency domain features (PASA slope [2–3 MHz], Midband fit [1–2 MHz], Midband fit [2–3 MHz], Midband fit [0–3 MHz], Intercept [2–3 MHz], Intercept [0–3 MHz], FWHM [0–0.5 MHz], FWHM [1–1.5 MHz], FWHM [2.5–3 MHz], Prominence [0–0.5 MHz], Prominence [1.5–2 MHz], Prominence [2.5–3 MHz] and Frequency domain area) being mostly positively and negatively correlated with the blood parameters, respectively.

**Fig 13:**
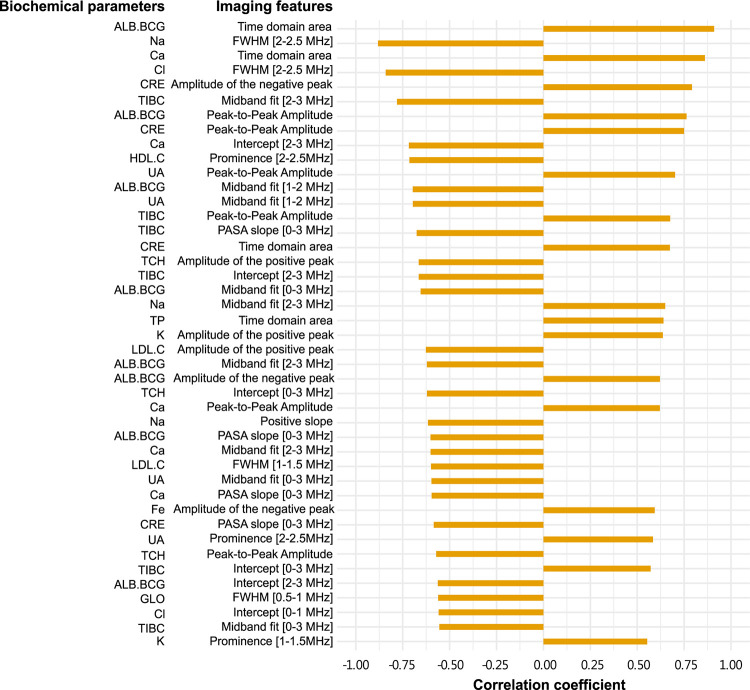
The correlation ranking between biochemical parameters and imaging features in photoacoustic system. Only significantly correlated features are shown (p–value≤0.05, r_s_ > 0.5). ALB.BCG (albumin), Ca, Cl, CRE (creatinine), Fe, GLO (globulin), HDL.C (high–density lipoprotein), K, LDL.C (low–density lipoprotein), Na, TCH (total cholesterol), TIBC (total iron binding capacity), TP (total protein), UA (uric acid).

**Fig 14:**
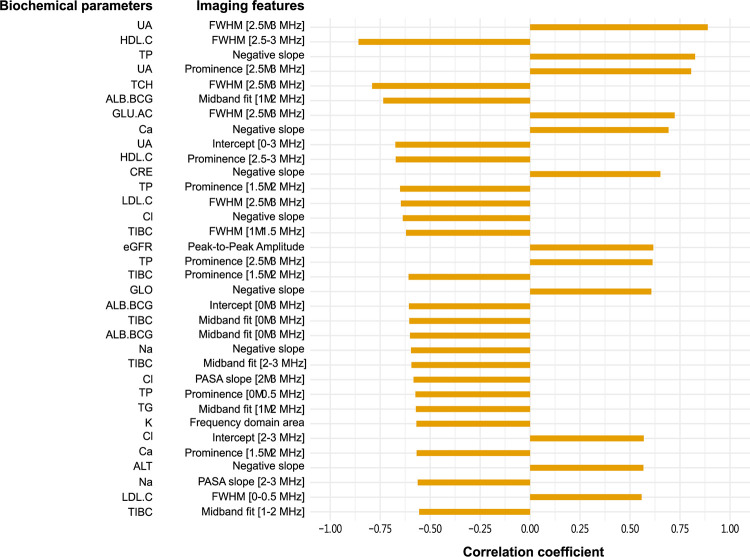
The correlation ranking between biochemical parameters and imaging features in absorption spectroscopy system. Only significantly correlated features are shown (p–value≤0.05, r_s_ > 0.5). ALB.BCG (albumin), ALT (alanine aminotransferase), Ca, Cl, CRE (creatinine), eGFR (estimated glomerular filtration rate), GLO (globulin), GLU.AC (glucose), HDL.C (high–density lipoprotein), K, LDL.C (low–density lipoprotein), Na, TCH (total cholesterol), TG (triglyceride), TIBC (total iron binding capacity), TP (total protein), UA (uric acid).

Blood urea nitrogen (BUN) was the only biochemical parameter that did not show any correlation to imaging features in both photoacoustic and absorption spectroscopy systems. It is interesting to note that the photoacoustic and absorption spectroscopy imaging detection methods are complementary: estimated glomerular filtration rate (eGFR), glucose (GLU.AC), triglyceride (TG) and alanine aminotransferase (ALT) were not significantly associated with photoacoustic imaging features, while Fe was not significantly associated with absorption spectroscopy imaging features. Upon ranking imaging features according to their correlation with the biochemical parameters we found that in the photoacoustic system albumin (ALB.BCG) and time domain area had highest Spearman’s correlation coefficient (r_s_ = 0.909) (Fig 13), while uric acid (UA) and FWHM [2.5–3 MHz] had the highest Spearman’s correlation coefficient (r_s_ = 0.888) in absorption spectroscopy system (Fig 14).

### Model building

The total of 14 individual biochemical parameters significantly correlated to photoacoustic imaging features (ALB.BCG, Ca, Cl, CRE, Fe, GLO, HDL.C, K, LDL.C, Na, TCH, TIBC, TP and UA, see Fig 13) as well as 17 individual biochemical parameters significantly correlated to absorption spectroscopy imaging features (ALB.BCG, ALT, Ca, Cl, CRE, eGFR, GLO, GLU.AC, HDL.C, K, LDL.C, Na, TCH, TG, TIBC, TP and UA, see Fig 14) were used to construct statistical models. Multicollinear features were eliminated using the step-wise approach described in *Methods*. If the variance inflation factor (VIF) of some features was above 10, the feature with the maximum VIF value was removed and the procedure repeated until all VIF values were smaller or equal 10 (S1 and S2 Tables). Multicollinearity analysis was not performed when there was no significant correlation between imaging features and biochemical parameters or when a prediction model was based on only one imaging feature. In the S1 and S2 Tables, “NA” indicates that the multicollinearity analysis was not conducted for the model. For the remaining imaging features, we determined whether they had a linear or a non-linear relationship with the blood parameters based on the linear regression diagnosis (S3 and S4 Tables). Both for linear and non-linear relationships, the optimal degree was identified based on the Bayesian information criterion (BIC). These parameters were used to construct the biomedical prediction models by polynomial regression.

**Fig 15:**
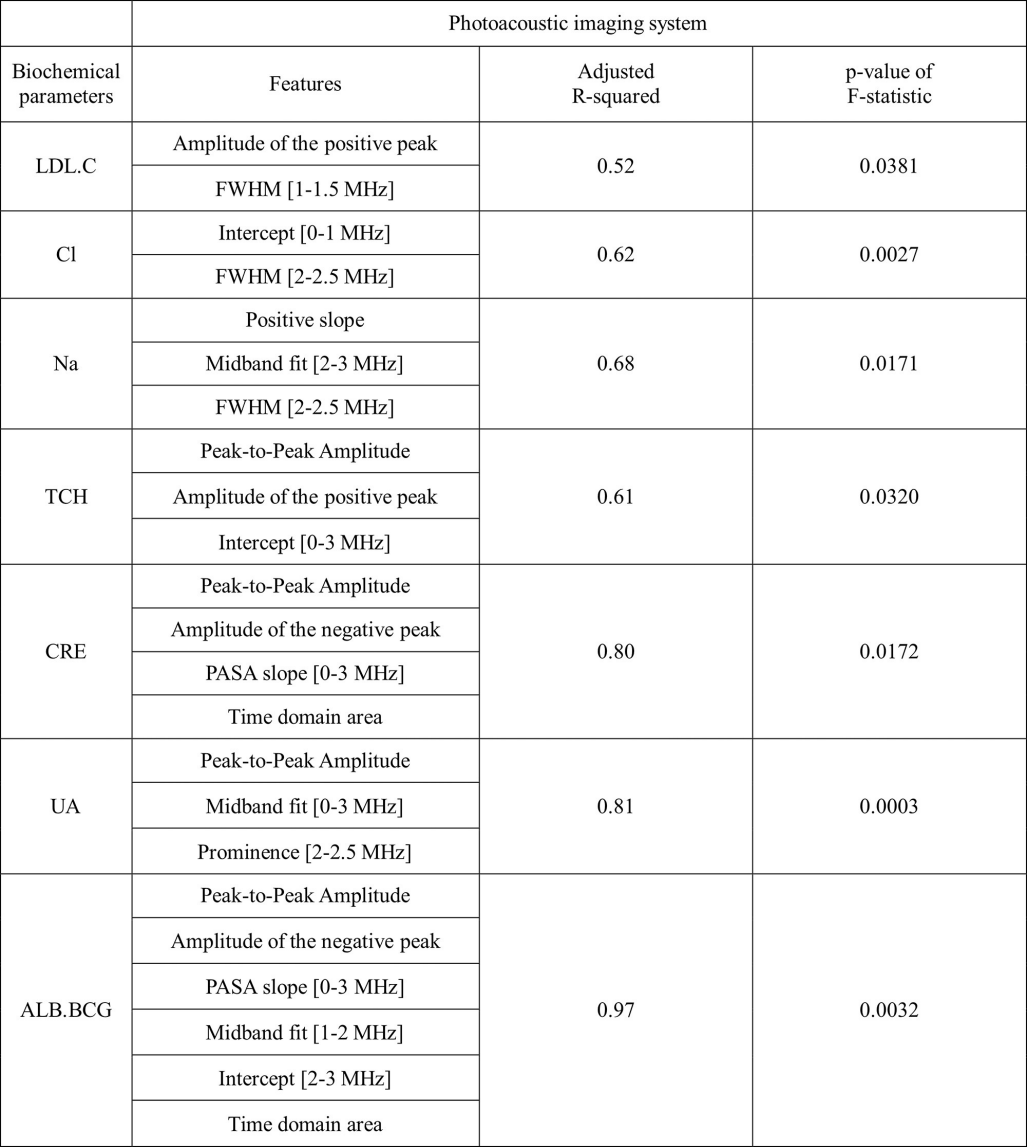
Polynomial regression models for the photoacoustic imaging system with better prediction. Only better prediction models are shown (adjusted R–squared values>0.5, p–values of F–statistic < 0.05). LDL.C (low–density lipoprotein), Cl, Na, TCH (total cholesterol), CRE (creatinine), UA (uric acid), ALB.BCG (albumin).

**Fig 16:**
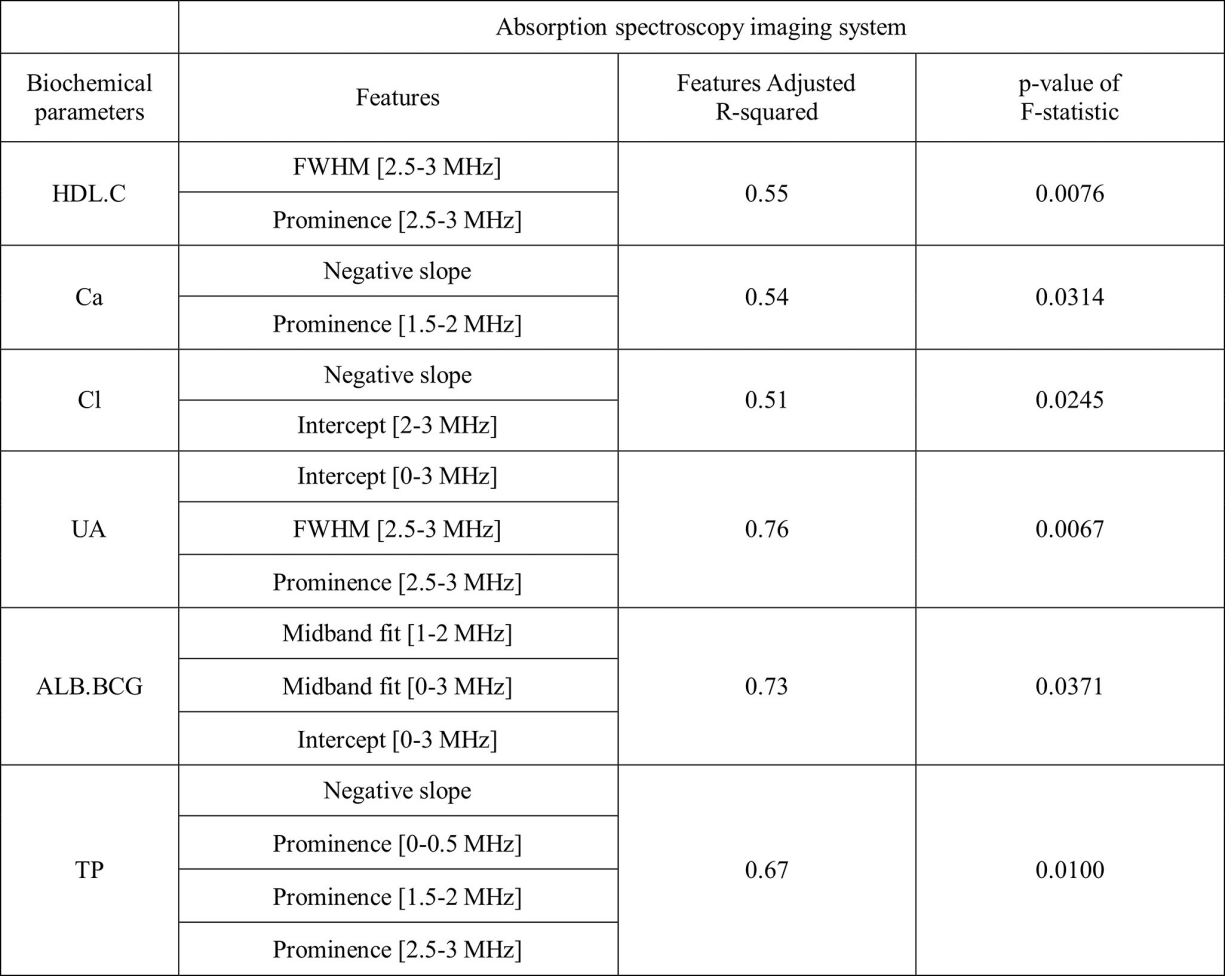
Polynomial regression models for the absorption spectroscopy imaging system with better prediction. Only better prediction models are shown (adjusted R–squared values>0.5, p–values of F–statistic < 0.05). HDL.C (high–density lipoprotein), Ca, Cl, UA (uric acid), ALB.BCG (albumin), TP (total protein).

For the photoacoustic imaging features, we identified seven polynomial regression models with an adjusted R-squared values higher than 0.5 and significant p-values of F-statistic (<0.05) involving the following biochemical parameters: LDL.C, Cl, Na, TCH, CRE, UA and ALB.BCG (Fig 15 and S3 Table). Interestingly, five of parameters (LDL.C, TCH, CRE, UA and ALB.BCG) were significantly correlated with the amplitude-related features (Peak-to-Peak Amplitude, Amplitude of the positive peak and Amplitude of the negative peak). CRE, UA and ALB.BCG appear to be especially well explainable by polynomial regression models as they have very high adjusted R-squared values (0.80, 0.81 and 0.97, respectively). For the absorption spectroscopy imaging features, we identified six significant polynomial regression models with the adjusted R-squared values >0.5 and p-values of F-statistic <0.05 involving the following biochemical parameters: HDL.C, Ca, Cl, UA, ALB.BCG and TP (Fig 16 and S4 Table), whereby the adjusted R-squared values of UA and ALB.BCG were higher than 0.7.

We found that UA and ALB.BCG can be accurately predicted by polynomial regression models constructed both for the photoacoustic and the absorption spectroscopy system (adjusted R-squared > 0.7 and p < 0.05) while CRE was only associated with a model for photoacoustic imaging features (R-squared = 0.83). Furthermore, although Fe, eGFR, GLU.AC, TG and ALT had significant correlation with photoacoustic or absorption spectroscopy imaging features, they could not be well explained by polynomial regression models. BUN had no significant correlation with either photoacoustic or absorption spectroscopy imaging features.

## Discussion and conclusions

Previous research demonstrated the ability of the photoacoustic imaging system to extract tissue architecture, optical contrast, and molecular distribution based on the optical absorption characteristics of chromophores, such as hemoglobin, melanin, water or lipids [53]. Photoacoustic signals have been applied in many blood-related fields, such as probing the size and shape of red blood cells [6, 7] and measuring blood glucose concentration [10]. However, so far the full spectrum of biochemical blood parameters has not been explored by imaging techniques.

Furthermore, in our research, we utilized a 0.5 ms pulse width, which is larger than usual (10 ns). Generally, low pulse widths are used to detect the tissue texture. Meanwhile, to avoid the influence of the plastic tube on the propagating acoustic wave, we utilized a large pulse width and the laser was focused on the tip position of the plastic containers to excite temperature change A previous study has shown the enhancement of PA signals as the laser pulse width increased. At the same time, the temperature of the focal point also increased [54]. In the absorption spectroscopy imaging experiment, we compared the reference and the probe beam produced by the light passing through the blood sample and water, respectively, thus mitigating the influence of the plastic tube on the signal.

In this study, we investigated the extent to which time and frequency domain features derived from photoacoustic and absorption spectroscopy imaging signals reflect the values of biochemical blood parameters. Our results indicate that a number of imaging features are indeed informative in this respect and can therefore be potentially useful in the clinical diagnosis. We have also demonstrated that linear and non-linear combinations of photoacoustic/absorption spectroscopy imaging features can accurately predict some of the biochemical blood parameters. However, there are still some biochemical parameters that cannot be well predicted. In future studies, we intend to test different wavelengths for analyzing photoacoustic/absorption spectroscopy features of blood.

Another obvious limitation of the presented report is that it is based on a small number of samples (drawn from 14 individuals). We anticipate that future studies involving more samples will be able to achieve higher prediction accuracy. In addition, the same approach could also be applied to saliva analysis since most of the compounds found in blood are also present in saliva [55]. It would be very interesting to compare the results of the prediction models trained on blood and saliva samples. Finally, imaging-based technologies could be used for in vivo tests, thus reducing the need for conventional blood biopsies.

## Supporting information

S1 Table Multicollinearity analysis for the photoacoustic imaging system. NA, no multicollinearity analysis was performed.
(PDF)

S2 Table Multicollinearity analysis for the absorption spectroscopy imaging system. NA, no multicollinearity analysis was performed.
(PDF)

S3 Table Polynomial regression models for the photoacoustic imaging system. (PDF)

S4 Table Polynomial regression models for the absorption spectroscopy imaging system. (PDF)
