# Supplementary material for: Photoacoustic and absorption spectroscopy imaging analysis of human blood
Source: PLoS One. 2023 Aug 4;18(8):e0289704. doi: 10.1371/journal.pone.0289704 (PMC10403132; doi:10.1371/journal.pone.0289704)
Supplement: S1 Table — NA, no multicollinearity analysis was performed. (PDF) [file pone.0289704.s001.pdf]

S1 Table. Multicollinearity analysis for the photoacoustic system. NA, no multicollinearity analysis was performed.

Photoacoustic imaging system						
	Step 1		Step 2		Step 3	
**Biochemical parameters**	Feature	VIF	Feature	VIF	Feature	VIF
**LDL.C**	Amplitude of the positive peak	1.00				
	FWHM [1-1.5 MHz]	1.00				
**HDL.C**	Prominence [2-2.5 MHz]	NA				
**TIBC**	Peak-to-Peak Amplitude	2.86	Peak-to-Peak Amplitude	2.86	Peak-to-Peak Amplitude	1.35
	PASA slope [0-3 MHz]	4.41E+08	Midband fit [2-3 MHz]	349.88	Midband fit [0-3 MHz]	1.50
	Midband fit [2-3 MHz]	359.36	Midband fit [0-3 MHz]	240.98	Intercept [2-3 MHz]	2.07
	Midband fit [0-3 MHz]	1.78E+08	Intercept [2-3 MHz]	6.55	Intercept [0-3 MHz]	1.67
	Intercept [2-3 MHz]	6.73	Intercept [0-3 MHz]	57.13		
	Intercept [0-3 MHz]	2.31E+08				
**Fe**	Amplitude of the negative peak	NA				
**Ca**	Peak-to-Peak Amplitude	1.78	Peak-to-Peak Amplitude	1.37		
	PASA slope [0-3 MHz]	11.52	PASA slope [0-3 MHz]	2.54		
	Midband fit [2-3 MHz]	15.91	Intercept [2-3 MHz]	2.36		
	Intercept [2-3 MHz]	2.36	Time-domain area	2.28		
	Time-domain area	4.36				
**Cl**	Intercept [0-1 MHz]	1.03				

	FWHM [2-2.5 MHz]	1.03		
**K**	Amplitude of the positive peak	1.48		
	Prominence [1-1.5 MHz]	1.48		
**Na**	Positive slope	1.25		
	Midband fit [2-3 MHz]	1.05		
	FWHM [2-2.5 MHz]	1.19		
**eGFR**	NA			
**GLU.AC**	NA			
**TG**	NA			
**TCH**	Peak-to-Peak Amplitude	1.61		
	Amplitude of the positive peak	1.47		
	Intercept [0-3 MHz]	1.26		
**CRE**	Peak-to-Peak Amplitude	5.39		
	Amplitude of the negative peak	4.57		
	PASA slope [0-3 MHz]	2.15		
	Time-domain area	1.99		
**UA**	Peak-to-Peak Amplitude	1.34	Peak-to-Peak Amplitude	1.09
	Midband fit [1-2 MHz]	26.85	Midband fit [0-3 MHz]	1.23
	Midband fit [0-3 MHz]	21.32	Prominence [2-2.5 MHz]	1.18
	Prominence [2-2.5 MHz]	2.07		
**BUN**	NA			
**GLO**	FWHM [0.5-1 MHz]	NA		

**ALB.BCG**	Peak-to-Peak Amplitude	8.81	Peak-to-Peak Amplitude	8.71	Peak-to-Peak Amplitude	7.18
	Amplitude of the negative peak	7.44	Amplitude of the negative peak	6.75	Amplitude of the negative peak	6.54
	PASA slope [0-3 MHz]	398.95	PASA slope [0-3 MHz]	3.33	PASA slope [0-3 MHz]	3.00
	Midband fit [1-2 MHz]	75.47	Midband fit [1-2 MHz]	19.94	Midband fit [1-2 MHz]	3.70
	Midband fit [2-3 MHz]	1373.01	Midband fit [0-3 MHz]	23.06	Intercept [2-3 MHz]	3.75
	Midband fit [0-3 MHz]	673.39	Intercept [2-3 MHz]	3.86	Time-domain area	4.98
	Intercept [2-3 MHz]	15.85	Time-domain area	5.26		
	Time-domain area	5.47				
**TP**	Time-domain area	NA				
**ALT**	NA					
