# Supplementary material for: Photoacoustic and absorption spectroscopy imaging analysis of human blood
Source: PLoS One. 2023 Aug 4;18(8):e0289704. doi: 10.1371/journal.pone.0289704 (PMC10403132; doi:10.1371/journal.pone.0289704)
Supplement: S2 Table — NA, no multicollinearity analysis was performed. (PDF) [file pone.0289704.s002.pdf]

S2 Table. Multicollinearity analysis for the absorption spectroscopy imaging system. NA, no multicollinearity analysis was performed.

Absorption spectroscopy imaging system				
	Step 1		Step 2	
**Biochemical parameters**	Feature	VIF	Feature	VIF
**LDL.C**	FWHM [0-0.5 MHz]	1.47		
	FWHM [2.5-3 MHz]	1.47		
**HDL.C**	FWHM [2.5-3 MHz]	2.68		
	Prominence [2.5-3 MHz]	2.68		
**TIBC**	Midband fit [1-2 MHz]	8.45	Midband fit [1-2 MHz]	3.08
	Midband fit [2-3 MHz]	3.82	Midband fit [2-3 MHz]	1.65
	Midband fit [0-3 MHz]	13.41	FWHM [1-1.5 MHz]	2.87
	FWHM [1-1.5 MHz]	2.90	Prominence [1.5-2 MHz]	1.13
	Prominence [1.5-2 MHz]	1.14		
**Fe**	NA			
**Ca**	Negative slope	1.17		
	Prominence [1.5-2 MHz]	1.17		
**Cl**	Negative slope	1.25	Negative slope	1.09
	PASA slope [2-3 MHz]	16.67	Intercept [2-3 MHz]	1.09
	Intercept [2-3 MHz]	15.68		
**K**	Frequency-domain area	NA		
**Na**	Negative slope	1.15		
	PASA slope [2-3 MHz]	1.15		
**eGFR**	Peak-to-Peak Amplitude	NA		
**GLU.AC**	FWHM [2.5-3 MHz]	NA		
**TG**	Midband fit [1-2 MHz]	NA		

**TCH**	FWHM [2.5-3 MHz]	NA
**CRE**	Negative slope	NA
**UA**	Intercept [0-3 MHz]	1.89
	FWHM [2.5-3 MHz]	2.69
	Prominence [2.5-3 MHz]	3.68
**BUN**	NA	
**GLO**	Negative slope	NA
**ALB.BCG**	Midband fit [1-2 MHz]	5.66
	Midband fit [0-3 MHz]	6.07
	Intercept [0-3 MHz]	2.12
**TP**	Negative slope	1.67
	Prominence [0-0.5 MHz]	1.54
	Prominence [1.5-2 MHz]	1.47
	Prominence [2.5-3 MHz]	1.63
**ALT**	Negative slope	NA
