# Supplementary material for: Photoacoustic and absorption spectroscopy imaging analysis of human blood
Source: PLoS One. 2023 Aug 4;18(8):e0289704. doi: 10.1371/journal.pone.0289704 (PMC10403132; doi:10.1371/journal.pone.0289704)
Supplement: S3 Table — (PDF) [file pone.0289704.s003.pdf]

S3 Table. Polynomial regression models for the photoacoustic imaging system.

	Photoacoustic imaging system				
Biochemical parameters	Features	Linear or Non-linear	Final degree	Adjusted R-squared	p-value of F-statistic
LDL.C	Amplitude of the positive peak	Non-linear	3	0.52	0.0381 *
	FWHM [1-1.5 MHz]	Linear	1		
HDL.C	Prominence [2-2.5 MHz]	Linear	1	0.48	0.0050 *
TIBC	Peak-to-Peak Amplitude	Linear	1	0.24	0.1939
	Midband fit [0-3 MHz]	Linear	1		
	Intercept [2-3 MHz]	Linear	1		
	Intercept [0-3 MHz]	Linear	1		
Fe	Amplitude of the negative peak	Linear	1	0.26	0.0420 *
Ca	Peak-to-Peak Amplitude	Non-linear	2	0.60	0.0569
	PASA slope [0-3 MHz]	Linear	1		
	Intercept [2-3 MHz]	Linear	1		
	Time domain area	Non-linear	2		
Cl	Intercept [0-1 MHz]	Linear	1	0.62	0.0027 *
	FWHM [2-2.5 MHz]	Linear	1		
K	Amplitude of the positive peak	Linear	1	0.28	0.0734
	Prominence [1-1.5 MHz]	Linear	1		
Na	Positive slope	Linear	1	0.68	0.0171 *
	Midband fit [2-3 MHz]	Non-linear	2		
	FWHM [2-2.5 MHz]	Non-linear	2		
TCH	Peak-to-Peak Amplitude	Linear	1	0.61	0.0320 *
	Amplitude of the positive peak	Non-linear	3		

	Intercept [0-3 MHz]	Linear	1		
CRE	Peak-to-Peak Amplitude	Linear	1	0.80	0.0172 *
	Amplitude of the negative peak	Linear	1		
	PASA slope [0-3 MHz]	Linear	2		
	Time domain area	Non-linear	3		
UA	Peak-to-Peak Amplitude	Linear	1	0.81	0.0003 *
	Midband fit [0-3 MHz]	Linear	1		
	Prominence [2-2.5 MHz]	Linear	1		
GLO	FWHM [0.5-1 MHz]	Non-linear	2	-0.08	0.6165
ALB.BCG	Peak-to-Peak Amplitude	Non-linear	2	0.97	0.0032 *
	Amplitude of the negative peak	Non-linear	3		
	PASA slope [0-3 MHz]	Linear	1		
	Midband fit [1-2 MHz]	Linear	1		
	Intercept [2-3 MHz]	Linear	1		
	Time domain area	Linear	1		
TP	Time domain area	Non-linear	2	0.15	0.1682

R^2, the coefficient of determination. * $p< .05$.
