# Supplementary material for: Photoacoustic and absorption spectroscopy imaging analysis of human blood
Source: PLoS One. 2023 Aug 4;18(8):e0289704. doi: 10.1371/journal.pone.0289704 (PMC10403132; doi:10.1371/journal.pone.0289704)
Supplement: S4 Table — (PDF) [file pone.0289704.s004.pdf]

S4 Table. Polynomial regression models for the absorption spectroscopy imaging system.

	Absorption spectroscopy imaging system				
Biochemical parameters	Features	Linear/Non-linear situation	Final degree	Features Adjusted R-squared	p-value of F-statistic
LDL.C	FWHM [0-0.5 MHz]	Non-linear	2	0.07	0.3270
	FWHM [2.5-3 MHz]	Linear	1		
HDL.C	FWHM [2.5-3 MHz]	Linear	1	0.55	0.0076 *
	Prominence [2.5-3 MHz]	Linear	1		
TIBC	Midband fit [1-2 MHz]	Linear	1	0.34	0.2503
	Midband fit [2-3 MHz]	Linear	1		
	FWHM [1-1.5 MHz]	Linear	3		
	Prominence [1.5-2 MHz]	Non-linear	2		
Ca	Negative slope	Non-linear	3	0.54	0.0314 *
	Prominence [1.5-2 MHz]	Linear	1		
Cl	Negative slope	Non-linear	2	0.51	0.0245 *
	Intercept [2-3 MHz]	Linear	1		
K	Frequency domain area	Linear	1	0.08	0.1786
Na	Negative slope	Non-linear	2	0.42	0.0490 *
	PASA slope [2-3 MHz]	Linear	1		
eGFR	Peak-to-Peak Amplitude	Non-linear	2	0.33	0.0533
GLU.AC	FWHM [2.5-3 MHz]	Non-linear	2	-0.04	0.4961
TG	Midband fit [1-2 MHz]	Linear	1	0.31	0.0289 *
TCH	FWHM [2.5-3 MHz]	Non-linear	2	0.47	0.0170 *
CRE	Negative slope	Non-linear	2	0.40	0.0299 *
UA	Intercept [0-3 MHz]	Linear	1	0.76	0.0067 *
	FWHM [2.5-3 MHz]	Non-linear	3		

	Prominence [2.5-3 MHz]	Linear	1		
GLO	Negative slope	Non-linear	2	0.21	0.1202
ALB.BCG	Midband fit [1-2 MHz]	Non-linear	2	0.73	0.0371 *
	Midband fit [0-3 MHz]	Non-linear	2		
	Intercept [0-3 MHz]	Non-linear	3		
TP	Negative slope	Linear	1	0.67	0.0100 *
	Prominence [0-0.5 MHz]	Linear	1		
	Prominence [1.5-2 MHz]	Linear	1		
	Prominence [2.5-3 MHz]	Linear	1		
ALT	Negative slope	Linear	1	0.08	0.1792

R^2, the coefficient of determination. * $p < .05$
